# Effective combination therapies in preclinical endocrine resistant breast cancer models harboring ER mutations

**DOI:** 10.18632/oncotarget.10852

**Published:** 2016-07-26

**Authors:** Brendon Ladd, Anne Marie Mazzola, Teeru Bihani, Zhongwu Lai, James Bradford, Michael Collins, Evan Barry, Anne U. Goeppert, Hazel M. Weir, Kelly Hearne, Jonathan G. Renshaw, Morvarid Mohseni, Elaine Hurt, Sanjoo Jalla, Haifeng Bao, Robert Hollingsworth, Corinne Reimer, Michael Zinda, Stephen Fawell, Celina M. D'Cruz

**Affiliations:** ^1^ Oncology iMed, AstraZeneca, Gatehouse Park, Waltham, MA, USA; ^2^ Oncology iMed, AstraZeneca, Alderley Park, Macclesfield, UK; ^3^ Oncology, Medimmune, Gaithersburg, MD, USA

**Keywords:** advanced metastatic breast cancer, ER mutations, endocrine resistance, combination therapy

## Abstract

Although endocrine therapy is successfully used to treat patients with estrogen receptor (ER) positive breast cancer, a substantial proportion of this population will relapse. Several mechanisms of acquired resistance have been described including activation of the mTOR pathway, increased activity of CDK4 and activating mutations in ER. Using a patient derived xenograft model harboring a common activating ER ligand binding domain mutation (D538G), we evaluated several combinatorial strategies using the selective estrogen receptor degrader (SERD) fulvestrant in combination with chromatin modifying agents, and CDK4/6 and mTOR inhibitors. In this model, fulvestrant binds WT and MT ER, reduces ER protein levels, and downregulated ER target gene expression. Addition of JQ1 or vorinostat to fulvestrant resulted in tumor regression (41% and 22% regression, respectively) though no efficacy was seen when either agent was given alone. Interestingly, although the CDK4/6 inhibitor palbociclib and mTOR inhibitor everolimus were efficacious as monotherapies, long-term delayed tumor growth was only observed when co-administered with fulvestrant. This observation was consistent with a greater inhibition of compensatory signaling when palbociclib and everolimus were co-dosed with fulvestrant. The addition of fulvestrant to JQ1, vorinostat, everolimus and palbociclib also significantly reduced lung metastatic burden as compared to monotherapy. The combination potential of fulvestrant with palbociclib or everolimus were confirmed in an MCF7 CRISPR model harboring the Y537S ER activating mutation. Taken together, these data suggest that fulvestrant may have an important role in the treatment of ER positive breast cancer with acquired ER mutations.

## INTRODUCTION

Breast cancer patients are subtyped based on the estrogen receptor (ER), progesterone receptor (PR), and/or ERBB2 (HER2) status of the tumor. The majority of patients presenting with ER+ disease can be successfully treated with endocrine therapies that block the production and/or activity of estrogen, thus depriving the tumor of its main driver for growth. Recommended hormonal depletion approaches depend on a women's menopausal status and may include treatment with aromatase inhibitors (AI), selective estrogen receptor modulators (SERM) and selective estrogen receptor degraders (SERD). Despite a multitude of clinical strategies to overcome estrogen signaling to the tumor, patients often become refractory and acquire endocrine resistant disease [1]. Recently, combination strategies of AIs with targeted agents for CDK4/6 (palbociclib) or mTOR (everolimus) have been approved for patients with metastatic disease. While the results from the combination approaches have been encouraging, the responses do not appear to extend overall survival. Although SERDs such as fulvestrant provide an additional endocrine strategy for reducing ER signaling, acquired resistance can emerge as well [2] and further reinforces the need to better understand mechanisms of resistance and evaluate combinatorial strategies.

Several mechanisms of endocrine resistance have been described including the overexpression or amplification of *HER2* [1–3] as well as mutations causing hyperactivation of the PI3K pathway [4, 5]. Recently, evidence of activating mutations in ER were described in tumors from patients with metastatic disease progressing on endocrine therapies [6–12]. To test the function of ER ligand binding domain (LBD) mutations *in vitro,* studies overexpressing a panel of ER LBD variants have demonstrated that ER mutations can promote ligand-independent activity and cellular growth [6–11]. Interestingly, Yu, *et al* generated cell lines from patient derived circulating tumor cells harboring recurrent mutations in *ER* and *PIK3CA* and performed an *ex vivo* compound screen [11]. The data demonstrated that SERDs can inhibit *in vitro* growth of these cell lines with the potential for more robust responses when used in combination with other targeted agents dependent on the genetic profile of the tumor. Unfortunately, cell lines with endogenous activating ER mutations are rare, limiting the ability to test *in vivo.* Patient derived xenograft (PDX) breast cancer models harboring ER mutations have recently been reported, and are useful tools for preclinical discovery. Li, *et al* described a PDX model harboring a Y537S ER mutation that recapitulated the estrogen independence observed in the patient from which the model was derived [6].

One strategy to block ligand-independent ER signaling is by inhibiting ER's function as a transcription factor by altering the chromatin state. To this end, it was recently demonstrated that JQ1, an inhibitor of the BET family of transcriptional regulators, suppressed ER activity and growth in tamoxifen-resistant cells [13]. Additionally, HDAC inhibition with vorinostat resensitized tamoxifen-resistant cells and resulted in synergistic growth inhibition with SERMs/SERDs [14]. In addition to ER mutation, activation of the mTOR pathway has been shown to promote acquired resistance to endocrine therapy [4], leading to the use of mTOR inhibitors such as everolimus in advanced breast cancer [15–17]. Indeed, while the BOLERO-2 trial reported promising results including increased progression-free survival when combining everolimus with an aromatase inhibitor [16, 17], there was no significant increase in overall survival [18]. Additionally, CDK4 has also been shown as a driver of estrogen independence [19] and the CDK4/6 inhibitor palbociclib selectively inhibits the growth of luminal ER+ cell lines [20, 21]. Given these observations, the PALOMA-1 trial evaluated the efficacy of palbociclib with an aromatase inhibitor and demonstrated an increase in progression free survival [22]. Collectively, these data warrant testing of SERDs with chromatin modifying agents and inhibitors of mTOR and CDK4 pathways in ER mutant breast cancer models.

In this report, we describe a CTX model (circulating tumor cell xenograft) with commonly co-occurring mutations including a recurrent ER mutation (D538G) [7, 9] that recapitulates clinically observed endocrine resistance. We demonstrate that this mutant ER protein is susceptible to degradation with fulvestrant. Despite this, the model remains only partially responsive to fulvestrant and insensitive to tamoxifen, potentially due to its complex genetic profile. The combination of fulvestrant with palbociclib or everolimus resulted in sustained tumor growth inhibition after treatment withdrawal and blocked the compensatory feedback observed with palbociclib or everolimus alone. Furthermore, while JQ1 or vorinostat alone altered binding of ER to chromatin and decreased target gene expression in the D538G background, these compounds only resulted in regressions when combined with fulvestrant. We also observed that all of the fulvestrant combinations decreased metastatic tumor burden. Finally, we tested fulvestrant in combination with palbociclib or everolimus in MCF7 cells with a different activating ER mutation (Y537S) engineered into the ligand binding domain using CRISPR. In this cell line, we again observed estrogen independent growth, partial sensitivity to fulvestrant, and an additive effect when combining fulvestrant with palbociclib or everolimus. Here, we describe clinically relevant models of ER mutant, endocrine resistant breast cancer and provide evidence that combination therapies including SERDs may benefit patients with activating ER mutations.

## RESULTS

### Characterization of endocrine resistant breast cancer by a CTX harboring an ER D538G mutation

**Figure 1:**
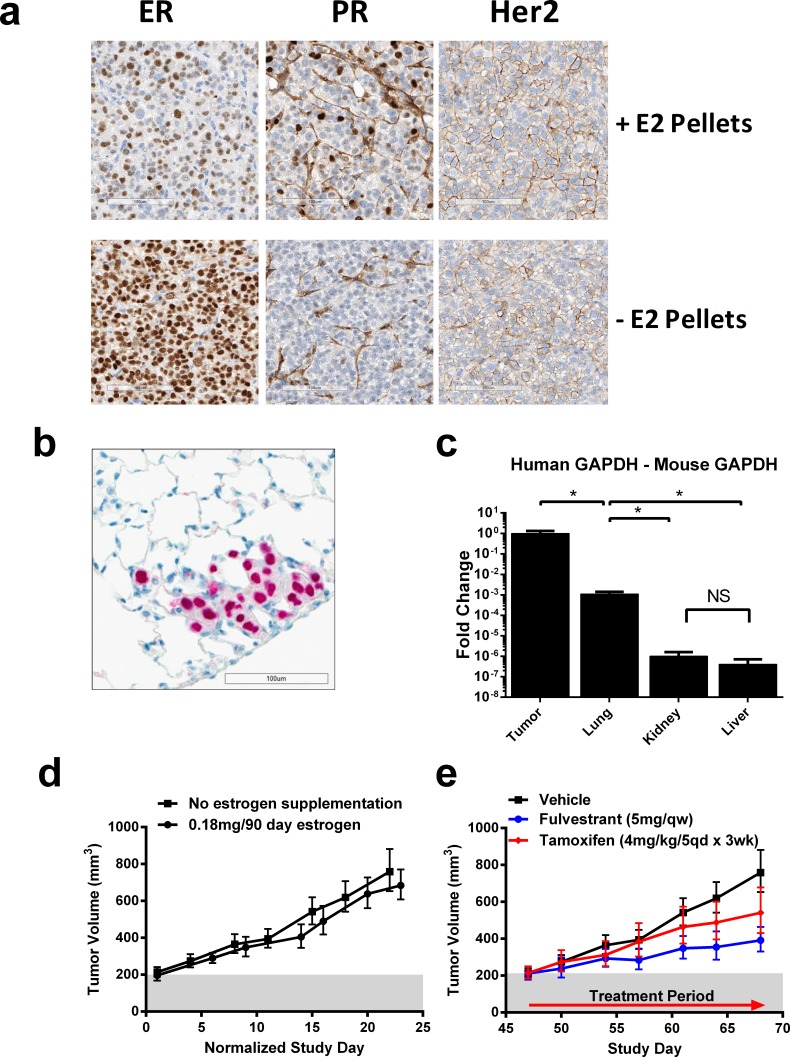
Human CTX model harboring D538G mutation recapitulates endocrine resistant, ER+ disease and metastasizes to the lungs **A.** Representative immunohistochemical staining of ER/PR/Her2 of tumors from CTC-174 tumor bearing mouse supplemented with estrogen pellets (0.18mg/90d) (top) and without estrogen supplementation (bottom). **B.** Human specific DNA-PKcs staining of a representative metastatic CTC-174 lung lesion. **C.** Detection of tumor tissue in tumor and mouse tissues using PCR for human and mouse GAPDH. Data represents the fold change of human GAPDH compared with mouse GAPDH. *N* = 3, * indicates *p* < 0.05. **D.** Tumor growth in vehicle treated animals +/− estrogen supplementation (0.18mg/90d). *N* = 7 animals. **E.** Efficacy of tamoxifen and fulvestrant in CTC-174 tumors. N≥7 animals. Bars represent SEM. Tamoxifen tumor growth inhibition (TGI) = 40.21%, *p* = 0.0546, Fulvestrant TGI = 66.54%, *p* = 0.0001.

We molecularly characterized a panel of 15 PDX models to elucidate the genomic mutations underpinning disease, five of which were ER+. Of the five models characterized, we identified one model, CTC-174, which harbors an ER D538G mutation, found at 31% allele frequency (Table S1). This model was generated from circulating tumor cells implanted orthotopically into immunocompromised mice (referred to as a CTX model). The circulating tumor cells were obtained from a 63-year-old patient with stage IV ER+ breast cancer after 42 days of fulvestrant therapy and 26 days of eribulin therapy. Although the patient had significant clinical improvement when the cells were collected, the patient presented with progressive metastatic disease 63 days later. Gene signature analyses suggested the model was representative of a luminal B subtype (Table S1, data not shown). Immunohistochemistry (IHC) of the implanted tumor confirmed this model was ER+/PR+/Her2 low, which was observed in the presence and absence of exogenous estrogen (Figure 1A). In addition, this model harbored other co-occurring oncogenic drivers including an activating PIK3CA mutation [7](Table S1).

To determine the metastatic potential of CTC-174 in mice, we analyzed lung, liver, and kidney for the presence of human cancer cells. By immunohistochemistry (IHC) analysis, human cells were detected in lung tissue, suggesting this model possessed the ability to metastasize in an animal model (Figure 1B, data not shown). We validated the IHC results with PCR and observed significantly more human GAPDH RNA in the lungs compared to other tissues, consistent with IHC results (Figure 1C). We did not detect metastases in the kidney or liver (data not shown). The metastatic potential of this model allows for evaluation of a clinically valuable secondary endpoint.

Next, we wanted to determine if this model was estrogen independent, similar to previously reported ER mutant (D538G) models. Although most ER+ PDX models are estrogen dependent and require exogenous estrogen for growth, CTC-174 displayed similar growth kinetics in the presence or absence of estrogen supplementation (Figure 1D) and exhibited constitutive ER activity without estrogen supplementation (Figure S1). Using this model without exogenous estrogen more accurately recapitulated the tumor environment from which the model was derived (a postmenopausal woman); and all subsequent *in vivo* studies were therefore performed in the absence of exogenous estrogen. We next wanted to evaluate sensitivity of the model to tamoxifen, a SERM that is the current standard of care for ER+ breast cancer. In mice bearing CTC-174 tumors, only modest tumor growth inhibition with tamoxifen treatment was observed (Figure 1E, S2A). As expected, we observed a partial tumor growth inhibition (TGI = 59%) with fulvestrant (Figure 1E, S2A) in CTC-174, consistent with the clinical outcome of the patient.

**Figure 2:**
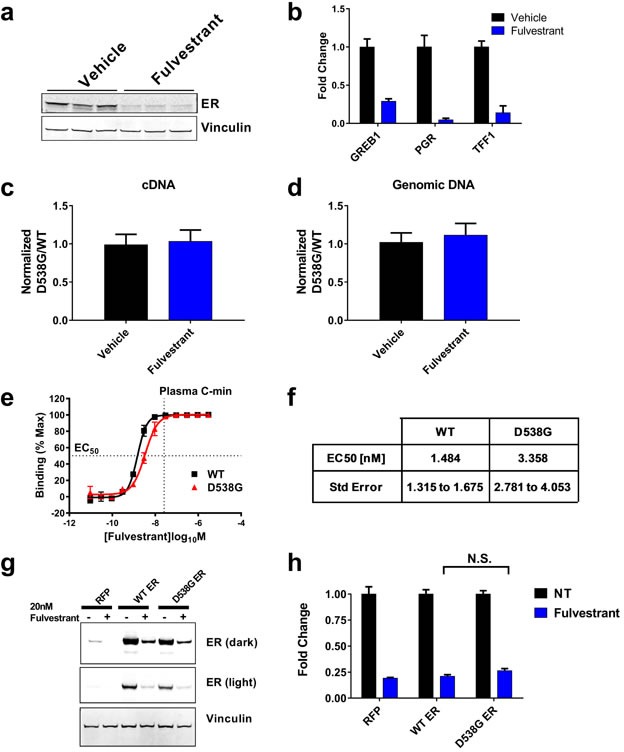
Fulvestrant can degrade the D538G mutant ER protein **A.** Western blot measuring ER protein levels from vehicle or fulvestrant treated animals at the end of the study (Figure 1D, *n* = 3 animals/treatment, Fulvestrant decreased ER 72.5%, *p* = 0.0004). GAPDH used as a loading control. **B.** PCR measuring ER target gene expression from vehicle or fulvestrant treated animals. **C.** Genotyping of ER cDNA. Data represented as the relative fold change of D538G ER over WT ER alleles relative to vehicle control. **D.** Genomic WT *vs*. D538G ER DNA represented as in **C.**. **E.** Fulvestrant binding to the ligand binding domain of WT or D538G ER measured by displacement of 1.5nM estrogen. Vertical line indicates the plasma C-min observed in the CONFIRM trial [25]. **F.** EC_50_ calculated from **E.** as described in the methods/materials. *p* = 9.79217E-14. **G.** Western blot analyses of the indicated proteins in MCF7 cells stably expressing a doxycycline inducible FLAG-WT or D538G ER treated with 20nM fulvestrant for 72 in the presence of 1ug/ml doxycycline. Vinculin was used as a loading control. **H.** Quantification of western blot from **G.** normalized to the respective untreated control. Histogram represents the average of 3 replicates. Bars represent SEM. N.S. = not significant (*p* = 0.2443)

To determine if the partial activity of fulvestrant was due to an altered ability to degrade mutant ER, we measured changes in ER protein levels and observed significant degradation (Figure 2A). Consistent with this, we observed downregulation of ER target gene expression (Figure 2B) suggesting fulvestrant is targeting the ER mutant protein as well as the WT ER allele. We hypothesized that the residual ER protein/activity observed was due to a reduced ability of fulvestrant to target the D538G ER protein and that treatment with fulvestrant would enrich for cells with high expression of the D538G ER allele. To test this, we used a PCR-based genotyping assay to measure the expression of the D538G *vs*. WT ER. Importantly, we did not observe a selection for cells with elevated expression of the D538G ER allele or increase in the abundance of the genomic D538G ER (Figure 2D). To further determine whether fulvestrant can target the D538G ER protein, we directly measured the ability of fulvestrant to displace 1.5nM estrogen from a GST-tagged ER ligand binding domain (LBD) of either the WT or D538G ER (amino acids 307-554) using time-resolved fluorescence resonance energy transfer (TR-FRET) (Figure 2E-2F). The results demonstrated that fulvestrant binds both the D538G-LBD and WT-LBD with low nanomolar affinity, and the EC_50_ of D538G-LBD was 2.26 fold higher than the EC_50_ of the WT-LBD (Figure 2F). Additionally, the EC_50_ towards both peptides was below the steady state plasma concentration of fulvestrant in patients, [23–25] suggesting fulvestrant should bind the D538G protein as well as WT and target them for degradation *in vivo*. To further test our hypothesis that fulvestrant would target mutant forms of ER, we generated MCF7 stable cell lines with a doxycycline inducible FLAG-WT or FLAG-D538G ER. After 72hrs of 20nM fulvestrant treatment, endogenous ER, FLAG-WT and FLAG-D538G ER proteins were degraded 80.6%, 79.0% and 73.5%, respectively and were not statistically different (Figure 2G-2H). Collectively, these data demonstrate that fulvestrant targets both the WT and D538G ER proteins for degradation and inhibits ER signaling, suggesting that the partial response observed *in vivo* is not due to an inability of fulvestrant to target the ER mutant protein.

### Efficacy of fulvestrant with JQ1, vorinostat, palbociclib or everolimus in the D538G ER CTX model

**Figure 3:**
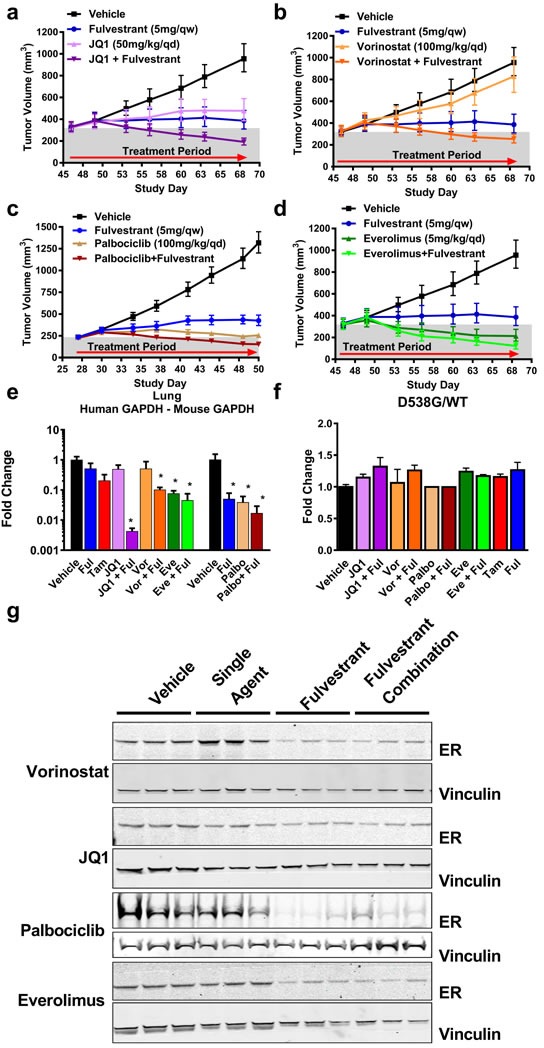
Efficacy of current therapeutic options with fulvestrant in a D538G ER background **A.**-**D.** Combination therapies with palbociclib (Palbo), vorinostat (Vor), JQ1 or everolimus (Eve) and fulvestrant (Ful) were performed in ovariectomized mice as indicated. 3A-B, 3D were performed in the same experiment and data was separated into individual graphs for clarity. *N* = 8. Palbociclib was tested separately and is represented with the matched vehicle and fulvestrant group. *N* = 10. Bars represent the SEM. Relative to vehicle-treated tumors: JQ1 TGI = 78%, *p* < 0.0001. JQ1+Ful = 41% regression, *p* < 0.0001. Vor TGI = 21%, *p* = 0.1189. Vor+Ful = 22% regression, *p* < 0.0001, Palbo TGI = 98%, *p* < 0.0001, Palbo+Ful = 30% regression, *p* < 0.0001. Eve = 33% regression, *p* < 0.0001. Eve+Ful = 62% regression, *p* < 0.0001. Ful for 3A-B, D TGI = 92%, *p* < 0.0001, 3C TGI = 83%, *p* < 0.0001. Relative to fulvestrant alone, Vor+Ful *p* = 0.0019. Eve *vs*. Eve+Ful *p* = 0.0075, Palbo *vs*. Palbo+Ful *p* = 0.0003 **E.** Detection of lung metastasis at the end of dosing from Figure, 3A-3D (*N* = 3). Samples were collected 24hr post fulvestrant and 4hr post-treatment for other agents. * *p* < 0.05 compared to vehicle. Tam = Tamoxifen **F.** Genotyping of WT ER *vs*. D538G ER in cDNA as in Figure 2E. No statistical significance was observed relative to the vehicle. **G.** Western blot measuring ER from 3 representative animals represented in 3A-D. Vinculin was used as a loading control.

Patients that have acquired endocrine resistance will progress to combination therapies that often include an endocrine agent. Because we have determined that fulvestrant is capable of targeting the D538G ER protein, including SERDs such as fulvestrant in these combination therapies may provide additional anti-tumor activity. We assessed the combination efficacy of fulvestrant with the chromatin modifying agents, JQ1 (BET family inhibitor), vorinostat (class I and II HDAC inhibitor), palbociclib (CDK4/6 inhibitor) and everolimus (TORC1 inhibitor). Importantly, all of these agents were well tolerated in combination with fulvestrant (data not shown). Treatment with JQ1 alone resulted in a 78% TGI, while vorinostat alone had little to no efficacy. Surprisingly, both JQ1 and vorinostat resulted in tumor regressions when combined with fulvestrant (Figure 3A-3B). Significant efficacy was observed with palbociclib or everolimus monotherapy (Figure 3C-3D). Although fulvestrant combination with palbociclib or everolimus were statistically different from the respective single agent group (*p* = 0.0003 and *p* = 0.0075, respectively), the efficacy observed with each agent alone made it difficult to conclude if the combination with fulvestrant was beneficial (Figure 3C-3D).

In addition to measuring efficacy by orthotopic tumor growth, we monitored tumor burden in the lung as a secondary endpoint. Animals treated with palbociclib or everolimus had significantly reduced lung tumor burden while the chromatin modifying agents JQ1 or vorinostat showed little effect compared to vehicle treatment (Figure 3E). Each combination group had significantly less lung tumor burden than vehicle treated animals (Figure 3E). Strikingly, fulvestrant in combination with JQ1 resulted in the most pronounced reduction of lung metastases despite the combination having a similar effect on primary tumors as other combinations. Metastatic lesions were also quantified by IHC staining of human DNA-PKcs in three random regions of the lung. The results further demonstrated that fulvestrant combinations decreased metastases compared to each respective agent alone (Figure S2B) suggesting that degrading ER in combination with other agents provides benefit for both primary and secondary endpoints.

To test if any agent provided a growth advantage for cells with increased D538G ER levels (relative to WT ER), we measured changes in the expression of the D538G and WT ER alleles following combination treatments (similar to Figure 2C-2D). None of the treatments resulted in a significant change in the ratio of the D538G and WT ER alleles, consistent with previous experiments (Figure 3F, S2C). In addition, none of the agents used in the combination groups altered the ability of fulvestrant to degrade ER (Figure 3G).

### Chromatin modifying agents alter ER function in the presence of an ER mutation

**Figure 4:**
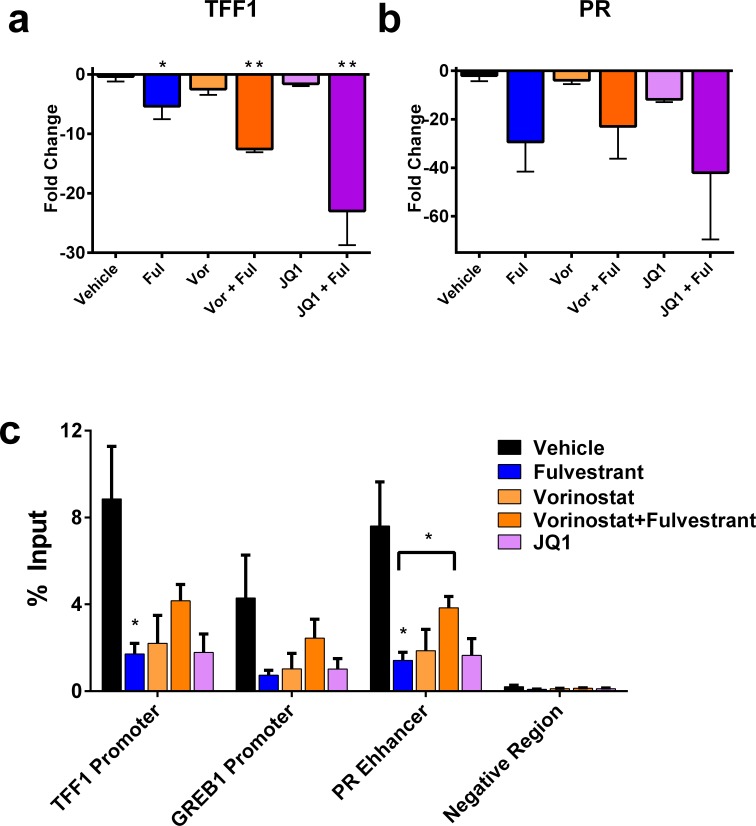
ER target gene expression and promoter/enhancer binding are decreased by the chromatin modifier inhibitors vorinostat and JQ1 **A.**-**B.** PCR of *TFF1* and *PR* from indicated animals in 3A, B. Each point represents the average of 3 animals, bars represent SEM. **C.** Chromatin immunoprecipitations (ChIP) was performed against ER in tumors represented in Figure 3. The ‘negative region’ is a negative control describing a genomic position downstream of the *PR* binding site. *p < 0.05, **p < 0.005 and is compared to vehicle unless otherwise indicated. Rabbit IgG are shown in Figure S2.

Previous studies have demonstrated that altering chromatin state may provide a mechanism of inhibiting ER. For example, the BET family inhibitor, JQ1, can decrease the expression of ER target genes such as *TFF1* in tamoxifen resistant cells [13]. Furthermore, it has been shown that HDACs associate with ER [26, 27], and are directly involved in the regulation of *TFF1* [28], and that HDAC inhibition can resensitize resistant cells to tamoxifen [29]. In the D538G ER mutant model, the chromatin modifying agents vorinostat and JQ1 decreased the expression of *TFF1*, and was augmented by combination with fulvestrant (Figure 4A). In addition to altering *TFF1* expression, both vorinostat and JQ1 decreased the expression of *PR* as well (Figure 4B). Finally, the decrease in ER target genes was specific to the chromatin modifying agents as palbociclib and everolimus did not result in decreases in ER target genes (*TFF1, PR and GREB1*) (Figure S3A).

Because vorinostat or JQ1 alone do not decrease ER protein levels, this suggests the altered ER target gene expression was a direct result of inhibiting ER function. To address this possibility, we performed chromatin immunoprecipitation (ChIP) of ER at the promoters and/or enhancer regions of ER target genes from tumor samples. Consistent with previous reports, fulvestrant treatment alone resulted in decreased ER binding to target genes [30] (Figure 4C). While vorinostat treatment slightly increased ER protein expression (Figure 3G), overall binding of ER to target genes was decreased (Figure 4C). As expected, the combination of vorinostat and fulvestrant resulted in a decrease in ER binding relative to the vehicle control (Figure 4C, S3B). Interestingly, the combination of vorinostat and fulvestrant trended towards increased ER occupancy at ER target gene promoters and enhancers compared to each single agent alone, while the expression of the target genes trended in the opposite direction. We also observed a decrease in ER occupancy after JQ1 treatment (Figure 4C), which is consistent with reports that JQ1 decreases the chromatin marks associated with active transcription in MCF7 cells at ER target genes [13]. We were unable to assess the occupancy of ER at target genes after JQ1 and fulvestrant treatment due to the consistent tumor regressions observed with this combination. Collectively, these data suggest that chromatin modifiers such as vorinostat or JQ1 enhance the efficacy of fulvestrant by indirectly modulating ER transcriptional activity.

### Compensatory feedback loops after palbociclib or everolimus treatment are mitigated by combination therapies

**Figure 5:**
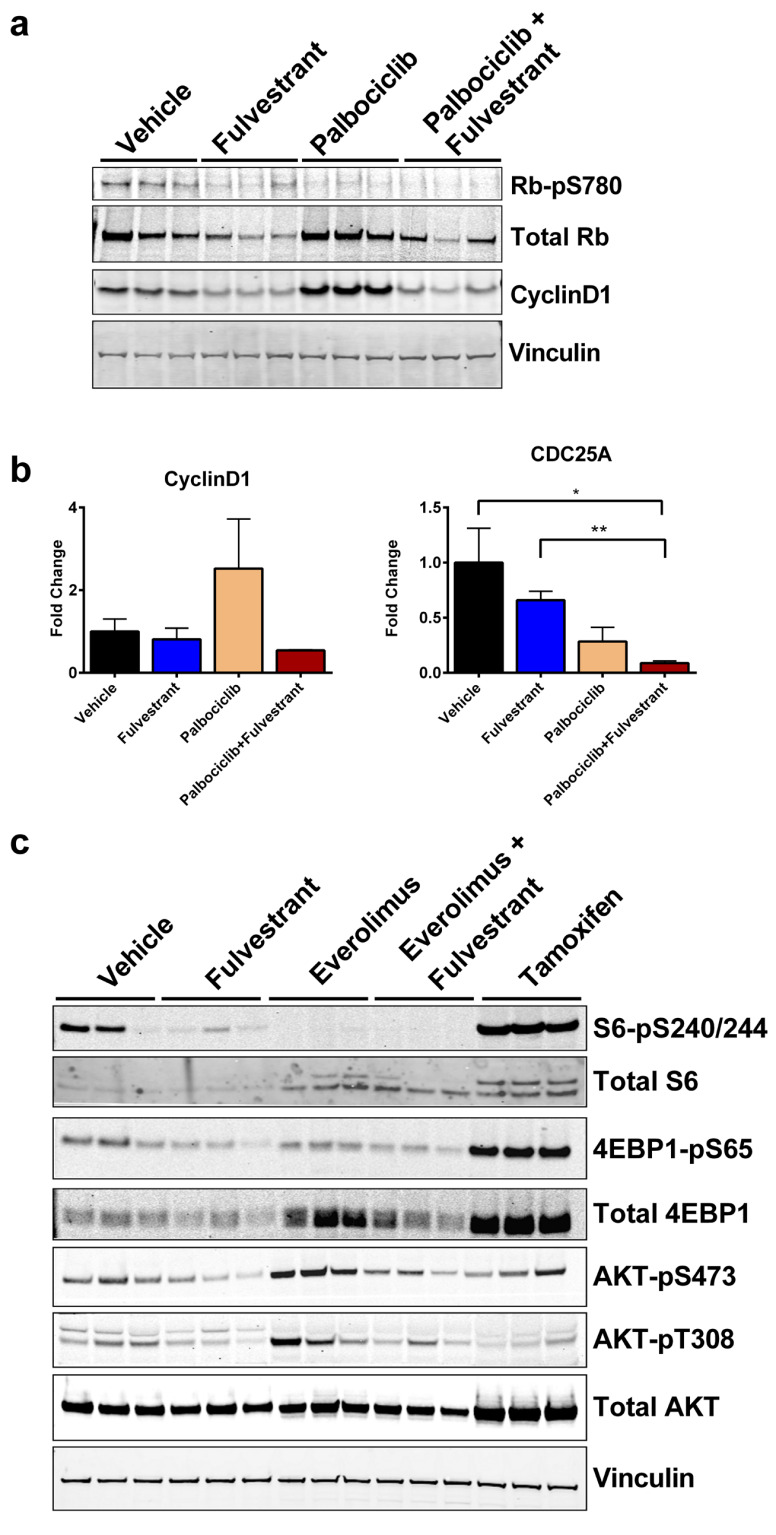
Feedback signaling from CDK4/6 (palbociclib) or mTOR (everolimus) inhibition is blocked by ER degradation in the D538G ER background **A.** Western blot of p780 RB and cyclinD1. **B.** Gene expression of *cyclinD1* (left) and *CDC25A* (right) from Figure 3C. Each point represents the average of 3 animals. Bars represent SEM. *p < 0.05, **p < 0.005 **C.** Western blot analyses of tumors from animals represented in Figure 3D. Vinculin was used as a loading control. Quantification displayed in Figure S3.

Previous studies demonstrated that treatment with palbociclib increased the protein levels of cyclinD1 [31, 32], which can promote ER activity independent of CDK4 [33–35]. Although the combination of palbociclib and fulvestrant was statistically better than palbociclib alone (Figure 3C), palbociclib was efficacious as a single agent. Therefore, we evaluated whether combining fulvestrant with palbociclib could provide a benefit by blocking the feedback signaling observed with palbociclib treatment [31]. First, we confirmed that palbociclib alone or in combination with fulvestrant decreased p780 Rb, an established biomarker of palbociclib activity [21] (Figure 5A). Consistent with these findings, we observed a significant increase in cyclinD1 protein levels with palbociclib (Figure 5A, S4A). Importantly, fulvestrant decreased cyclinD1 expression and blocked the increase observed with palbociclib when used in combination (Figure 5A). We also observed a similar trend with *cyclinD1* mRNA levels (Figure 5B). Furthermore, palbociclib resulted in a greater than additive decrease in *CDC25A*, a marker associated with cell cycle progression, when combined with fulvestrant (Figure 5B, right). Collectively, these results demonstrate that the combination of palbociclib with a SERD may benefit patients by blocking the compensatory signaling observed with palbociclib alone and further decrease the expression of genes required for cell cycle progression.

Similar to palbociclib, everolimus exhibited monotherapy response in the CTC-174 model. (Figure 3D). To determine whether there was a combination benefit with everolimus and fulvestrant, we evaluated several markers of mTOR and AKT activity post-treatment. As expected, everolimus treatment decreased pS6-S240/244 and p4EBP1-S65 (Figure 5C, S4B). Fulvestrant also decreased pS6-S240/244 and p4EBP1-S65, albeit to a lesser extent than everolimus (Figure 5C). Interestingly, tamoxifen increased total and phosphorylated S6 and 4EBP1 as well as total AKT (Figure 5C, S4B), highlighting the benefit ER degradation with fulvestrant compared to ER modulation with tamoxifen. Previous studies have demonstrated that inhibition of TORC1 with everolimus results in a compensatory increase in TORC2 mediated AKT activation and the abrogation of AKT signaling enhances the effects of everolimus inhibition [36–38]. We observed similar compensatory AKT activities in our model (Figure 5C). Interestingly, fulvestrant decreased pAKT-S473 and pAKT-T308 and blocked the everolimus-induced increase in pAKT (Figure 5C), suggesting a mechanistic benefit for combining fulvestrant with everolimus. Together, these results demonstrate ER degradation can enhance mTOR inhibition with everolimus in a D538G ER, mutant PIK3CA background.

### Concurrent inhibition of ER and CDK4/6 or mTOR leads to delayed tumor growth

**Figure 6:**
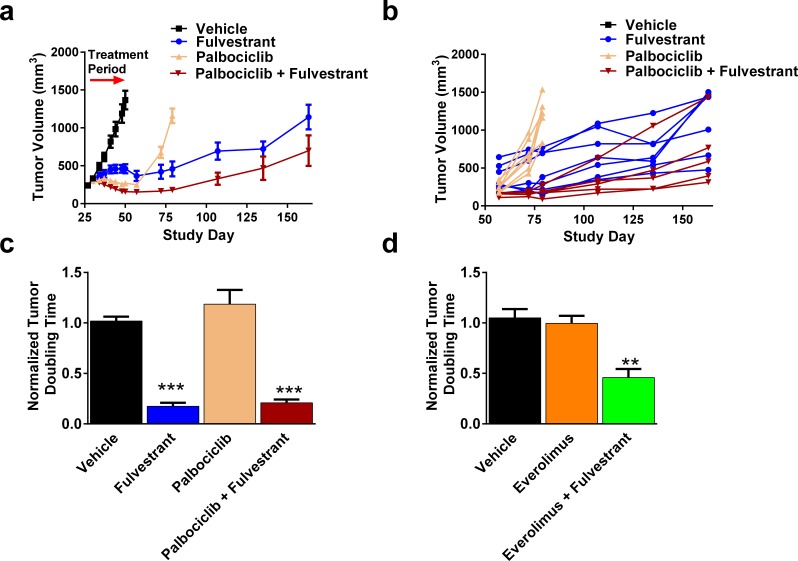
More durable responses with are achieved when palbociclib and everolimus are combined with fulvestrant **A.**-**B.** At least 5 animals from Figure 3C were allowed to regrow tumors after dosing with palbociclib alone or in combination with fulvestrant. **A.** Mean tumor volume. Arrow represents 21 dosing day period. **B.** Individual animal tumor growth starting from the end of dosing. **C.**-**D.** Tumor doubling times after ending treatment from animals treated with fulvestrant and/or palbociclib **C.** or everolimus **D.**. Bars represent SEM. ** *p* < 0.005, *** *p* < 0.0005.

Our data demonstrates that ER degradation can inhibit feedback signaling observed with palbociclib and everolimus and suggests that adding fulvestrant with either of these compounds may present a clinically beneficial strategy. To test this hypothesis further, we continued measuring tumor growth in groups where tumor regressions were observed. After discontinuing treatment with palbociclib or everolimus alone, tumors resumed growth at a similar rate as the vehicle treated animals (Figure 6A-6B, S5A-S5B), and was further confirmed by the quantification of tumor doubling time (Figure 6C-6D). Importantly, tumors from animals treated with fulvestrant alone or the fulvestrant/palbociclib combination had significantly delayed doubling time (5.71 and 4.77 fold slower relative to vehicle, respectively) (Figure 6C). Similarly, animals treated with fulvestrant and everolimus had significantly delayed tumor growth relative to vehicle (2.18 fold slower relative to vehicle) (Figure 6D). Collectively, fulvestrant alone or in combination with palbociclib or everolimus clearly delayed tumor regrowth and provides mechanistic rationale for evaluation in the clinic.

### Efficacy of fulvestrant with palbociclib or everolimus in an engineered MCF7 ER Y537S xenografts

**Figure 7:**
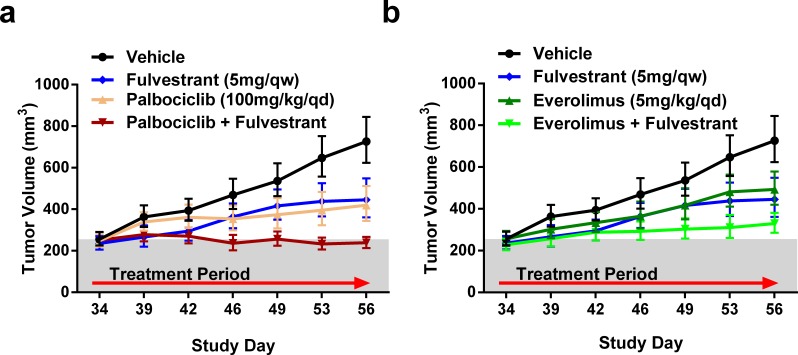
Efficacy of palbociclib or everolimus with fulvestrant in a MCF7-Y537S/KO background **A.**-**B.** Combination therapies of fulvestrant (Ful) with either palbociclib (Palbo) or everolimus (Eve) were performed in nude mice. All treatments were dosed in the same experiment and separated for clarity. *N* = 11. Bars represent SEM. Relative to vehicle: fulvestrant TGI = 52%, *p* = 0.0118, palbociclib TGI = 62%, *p* = 0.0032, Palbo+Ful = 5% regression, *p* < 0.0001, everolimus TGI = 50%, *p* = 0.0177, Eve+Ful TGI = 76%, *p* = 0.0002.

Our data in the ER mutant CTC-174 model suggests a benefit for combining fulvestrant with either palbociclib or everolimus. To confirm these observations in an additional model, we introduced several clinically relevant ER mutations into MCF7 cells using CRISPR/Cas9. These efforts yielded a polyclonal MCF7 cell line with a Y537S mutation in one ER allele and a frameshift mutation in another ER allele (ER-Y537S/KO). Consistent with the loss of an ER allele, the MCF7-Y537S/KO cell line has lower ER expression relative to the parental MCF7 cell line (Figure S6A). Additionally, when implanted in mice supplemented with estrogen pellets, we observed continuous tumor growth of MCF7-Y537S/KO cells *in vivo* after removing the estrogen pellets (Figure S6B), which is similar to previous overexpression studies [7]. We then assessed the efficacy of palbociclib or everolimus in combination with fulvestrant. Unlike CTC-174, no single agent treatments exhibited in tumor regressions (TGI of 52%, 50% and 62% for fulvestrant, everolimus and palbociclib, respectively) (Figure 7A-7B). The combination of palbociclib and fulvestrant resulted in a greater tumor growth inhibition (5% regression) than either agent alone, similar to previous reports in a PDX model with an ER-Y537S mutation [39]. The combination of everolimus and fulvestrant resulted in a 76% TGI suggesting the combination of fulvestrant and everolimus is additive in this model (Figure 7B). Together, these data provide a second ER mutant model demonstrating that the addition of fulvestrant to palbociclib and everolimus treatments will provide benefit in ER mutant breast cancers.

## DISCUSSION

ER+ breast cancers bearing activating ER mutations represent a new segment of endocrine resistant disease with an unmet therapeutic need. To investigate potential strategies to target these tumors, we developed an ER+ breast cancer CTX model from circulating tumor cells of a patient that harbors a D538G ER mutation, CTC-174. This mutation promotes estrogen independent ER activity and have been reported in patients who have acquired endocrine resistance [7, 9, 10, 40]. Indeed, our model recapitulates endocrine therapy resistant disease as shown by estrogen independent growth and resistance to tamoxifen. Using this model, as well as *in vitro* approaches, we demonstrated that fulvestrant targets the mutant ER protein for degradation but only provides modest growth inhibition *in vivo,* suggesting additional pathways may promote resistance to endocrine therapy.

Clinically, combinatorial strategies for AI refractory ER+ breast cancer have yielded encouraging results. The BOLERO-2 and PALOMA-1 trials both achieved increased progression free survival by combining an aromatase inhibitor with everolimus or palbociclib, respectively [16, 22]. Given that activating ER mutations are acquired most frequently in patients who have previously received an aromatase inhibitor [40], the combination of everolimus or palbociclib with a SERD such as fulvestrant may provide superior efficacy in these patients by lowering ER expression and could potentially increase overall survival [18]. Recently, the PALOMA-3 trial evaluating palbociclib combined with fulvestrant demonstrated longer progression free survival compared to fulvestrant alone [41]. Future follow-up with these patients may ultimately determine if this combination increases overall survival in patients with ER mutations. In support of this hypothesis, we demonstrate that our ER mutant models are estrogen independent and therefore unlikely to respond to an aromatase inhibitor and that fulvestrant is effective at degrading WT and MT forms of ER. Furthermore, we demonstrate that although everolimus and palbociclib are efficacious in the CTC-174 model (D538G ER, N345K PIK3CA), more potent tumor regressions were observed when these agents were combined with fulvestrant. In a second MCF7 model (Y537S ER, E545K PIK3CA), we observed increased tumor growth inhibition when fulvestrant was combined with palbociclib or everolimus. These observations suggest combinations with fulvestrant will benefit patients with estrogen independent breast cancers harboring a D538G or Y537S ER mutations and similar genetic drivers. These ER mutations are only two of several recently reported mutations [6–10], and therefore it will be important to test the ability of fulvestrant to target other ER mutations.

We also hypothesized that despite degradation of the mutant ER protein, the residual ER protein is sufficient to promote tumor growth and additional strategies will be necessary to achieve clinical benefit. Inhibiting ER transcriptional activity by changing chromatin state with chromatin modifying agents might therefore provide additional benefit. In support of this, we observed downregulation of the several ER target genes, upon treatment with chromatin modifying agents JQ1 and vorinostat. In some instances, (such as *TFF1*), combining chromatin modifying agents with fulvestrant resulted in stronger downregulation than either single agent, although this coincided with an unexplained trend towards increased ER occupancy at ER target gene promoters and enhancers compared to each single agent. Interestingly, vorinostat treatment resulted in decreased gene expression of both *TFF1* and *PR*, despite having minimal impact on tumor growth. Vorinostat has a short half-life [42, 43], therefore, may not produce sufficient exposure to result in tumor growth inhibition due to only transient inhibition of ER function. Additionally, combining fulvestrant with JQ1 resulted in substantial decreases in the metastatic tumor burden, which could be the result of increased ability to inhibit growth of tumor cells in the lung or extravasate from the orthotopically-implanted tumor. Future work will be required to test these possibilities. Collectively, these data suggest that developing new epigenetic agents that can inhibit ER activity is warranted.

In addition to causing tumor regression, all of our combination therapies decreased metastatic tumor burden. Metastases are the primary cause of death from solid tumors and present a common therapeutic challenge in patients [44]. Therefore, our CTX model provides a valuable tool for testing the impact of novel compounds on metastasis in future drug development.

In conclusion, many of the clinical trials currently enrolling endocrine resistant breast cancer patients will include an endocrine therapy in combination with new targeted therapies. Our data suggests including a SERD will provide superior efficacy to an aromatase inhibitor in the patients enrolled in these trials. Several oral SERDS have been developed and have yielded promising results [45–48]. Our results demonstrate that mutant ER can be targeted with fulvestrant and suggests that SERDs with activity against mutant ER should be prioritized. Collectively, our study indicates that degrading ER in an endocrine resistant, estrogen independent, ER mutant model can provide a therapeutic benefit and suggests that fulvestrant may have an important role in the treatment of ER positive breast cancer with acquired ER mutations.

## MATERIALS AND METHODS

### Generation of the CTX model/efficacy studies

Blood samples from MBC patients, consented according to the Human Biological Samples Policy, were purchased from Conversant Biologics. Due to patient confidentiality, minimal patient history was provided and previous endocrine therapy treatments are unknown. PBMCs were prepared using Lympholyte^®^-H (Cedar Lane Labs), EpCAM^+^CD44^+^ cells were isolated (manuscript in preparation), and placed into 6-well ultra-low attachment plates (Corning) with the Mammocult medium (Stem Cell Technologies). After 9 days in culture, cells were suspended in phosphate buffered saline (PBS), counted with a hemocytometer, and resuspended in PBS mixed with high concentration matrigel (BD Biosciences) at 10 mg/mL. Each aliquot of 0.2 mL containing 650 cells was orthotopically injected into the third mammary fat pad of three NOD/SCID (Cg-Prkdcscid Il2rgtm1Wjl/SzJ) (NSG) mice pre-implanted with estrogen pellets (0.36mg/90day). For efficacy studies with CTC-174, tumor fragments were implanted in the mammary fat pads of NSG mice (Jackson Labs) by standard techniques. For MCF7-Y537S/KO cells, nude mice (Taconic) were injected with 1×10^7^ cells subcutaneously in 1:1 PBS/Matrigel (Corning). Efficacy studies in MCF7-Y537S/KO cells were performed without estrogen supplementation. To measure estrogen independent growth, mice were pre-implanted with 0.72mg/90 day estrogen pellets (Innovative Research of America) subcutaneously using a 10g trocar 48 hours prior to cell implantation, as instructed by the manufacturer. Estrogen pellets were removed using standard surgical techniques when the MCF7-Y537S/KO group average was approximately 500mm^3^. When estrogen pellets were used in CTC-174, 0.18mg/90 day (Innovative research of America) were implanted at the time of orthotopic tumor implant. Animals were ovariectomized (by Jackson Labs) for combination efficacy studies (Figure 3). Doses of JQ1 [49, 50], vorinostat [51], palbociclib [19, 21] and everolimus [52, 53] were matched to clinically relevant exposures as previously described. Fulvestrant was administered weekly in a 100μl injection of clinical grade/formulation of fulvestrant at 50mg/ml. All analysis of tumor/lung samples at the end of dosing in Figures 3–5 were performed using 3 representative animals. All tumor doubling times reflect growth from the last day of dosing until the last measurement. A doubling time for every animal in the respective group was generated using a best-fit exponential curve using by non-linear regression then averaged (prism 6). Each point represents the average of at least 5 animals, bars represent SEM. Stars indicate a *p*-value < 0.05. All procedures were performed in accordance with federal, state and Institutional guidelines in an AAALAC-accredited facility and were approved by the MedImmune or AstraZeneca Institutional Animal Care and Use Committee (IACUC).

### RNA sequencing/CGH array

Transcriptome resequencing was performed on an Illumina HiSeq 2000. Raw sequences were first quality assessed and low quality reads were removed. The clean reads were then aligned to both human (hg19) and mouse (mm10) using TopHat. In-house customized analysis pipeline was used to separate human and mouse sequences using alignment qualities [54]. Mouse sequences were then filtered from human alignment before downstream analysis. Expression was quantified into RPKM using and RSeQC. Variants from RNA-seq were called by an in-house variant caller VarDict. Mutations were identified after filtering common SNPs and non-functional variants, such as silent and those in UTR or introns.

### Immunohistochemistry and chromatin immunoprecipitation

Tumors and lungs Immunohistochemistry was performed using the Ventana Discovery XT according to manufacturer's instructions. All ChIP experiments were performed as previously described [55] with the following modifications: tumors were snap frozen in liquid nitrogen, crushed to powder and resuspended in 8ml 1% formaldehyde at room temperature.

### Time-resolved fluorescence resonance energy transfer

GST-tev-WT or D538G (residues 307-554) were expressed in E. coli, purified by GST capture followed by size exclusion then ion exchange chromatography. Binding was measured with a LanthaScreen kit (Life Technologies).

### Gene expression and genotyping of ER

All gene expression was performed using Taqman reagents according to the manufacturer's instructions (ER #Hs00174860_m1, TFF1 #Hs00907239_m1, PGR #Hs01556702_m1, GAPDH #Hs02758991_g1, GREB1 #Hs00536409_m1, cyclinD1 #Hs00765553_m1, CDC25A #Hs00947994_m1). GAPDH for evaluating lungs was human #4352934e or mouse #4352932e. For genotyping of the D538G mutation, a custom CAST-PCR system was purchased from Life Technologies (SKU#/PPL 4476206, Quote# P1019473).

### Tissue culture

MCF7 (ATCC) were cultured in RPMI + L-glutamine (Gibco) with 10% FBS (Sigma) under normal conditions [56]. Stable cell lines were generated by lentiviral infection using standard techniques. ER vectors are in a pTRIPZ backbone and are doxycycline inducible. When evaluating ER expression, cells were plated with 1ug/ul doxycycline. 24hrs after plating, media was replaced with either fresh media containing 1ug/ul doxycycline either with or without 20nM fulvestrant. Cells were incubated for 72hrs in fulvestrant before collecting and western blotting according to standard techniques. MCF-7 cells were genetically engineered to knock-in the ER Y537S mutation under neomycin selection. After two week of selection single cell clones were isolated and characterized. To confirm the knock-in, a digital droplet PCR was performed [57] using ddPCR primers (CGGGTTGGCTCTAAAGTAGT and AATGCGATGAAGTAGAGCCC) and specific probes (BHQ_cc [C]ctc [tAt]gacc [t]g_HEX and BHQ_CC [A]CTC [TCT]GAC [C]TG_FAM). The location of the insertion was confirmed using junction PCR with the following primer pairs, 1 (TTAGATCATGCTGTAGGCCCTG) + 2 (CTGGAACCCATGACCGGAAAG), 3 (GCAGATCCAGGGGGCATTTA) + 4 (GATGTGGAATGTGTGCGAGC), 2 (CTGGAACCCATGACCGGAAAG) 5 (GGATCAATTCTCTAGAGCTCGC). Tide analysis [58] was used to confirm the frame shift mutation of the 2nd ER allele. To measure ER-pS118, MCF7 cells were plated in charcoal/dextran stripped serum (Hyclone) for three days with daily washing with PBS to remove any residual estrogen. Where indicated, media with 10nM estrogen was added 24hr before harvesting.

### Antibodies

Vinculin (Sigma #V4505), ER antibodies: Santa Cruz cat# sc543 or Millipore #04-820), ER pS118 (Cell Signalling #2511), ER for IHC (Ventana #790-4324 [SP1]), p308-AKT (Cell Signaling #2965), p473-AKT (Cell Signaling #4060), AKT (Cell Signaling #4691), p240/244 S6 (Cell signaling #5364), p65-4EBP1 (Cell Signaling #9456), 4EBP1 (Cell Signaling #9452), cyclinD1 (Cell Signaling #2926), Rabbit IgG (Sigma, I5006). IHC: PR (1E2) Her2 (4B5) (Ventana Discovery XT). Biotinylated secondary antibodies (Vector Labs PK-6101).

## SUPPLEMENTARY MATERIALS


