# Supplementary material for: Effective combination therapies in preclinical endocrine resistant breast cancer models harboring ER mutations
Source: Oncotarget. 2016 Jul 26;7(34):54120–36. doi: 10.18632/oncotarget.10852 (PMC5342331; doi:10.18632/oncotarget.10852)
Supplement: Supplementary file 1 [file oncotarget-07-54120-s001.pdf]

Effective combination therapies in preclinical endocrine resistant breast cancer models harboring ER mutations

Supplementary Material

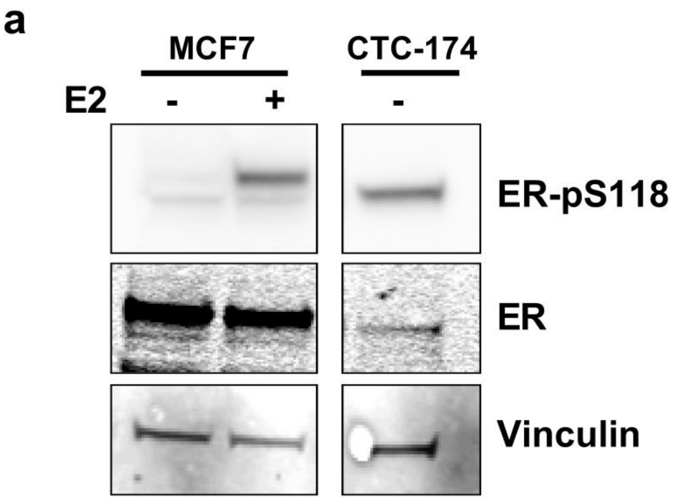

Supplementary Figure 1: Western blot showing ER-pS118 in CTC-174. MCF7 cells grown +/- estrogen used as positive/negative controls, respectively. All samples were blotted on the same membrane, shown at the same exposure level and cropped for clarity.

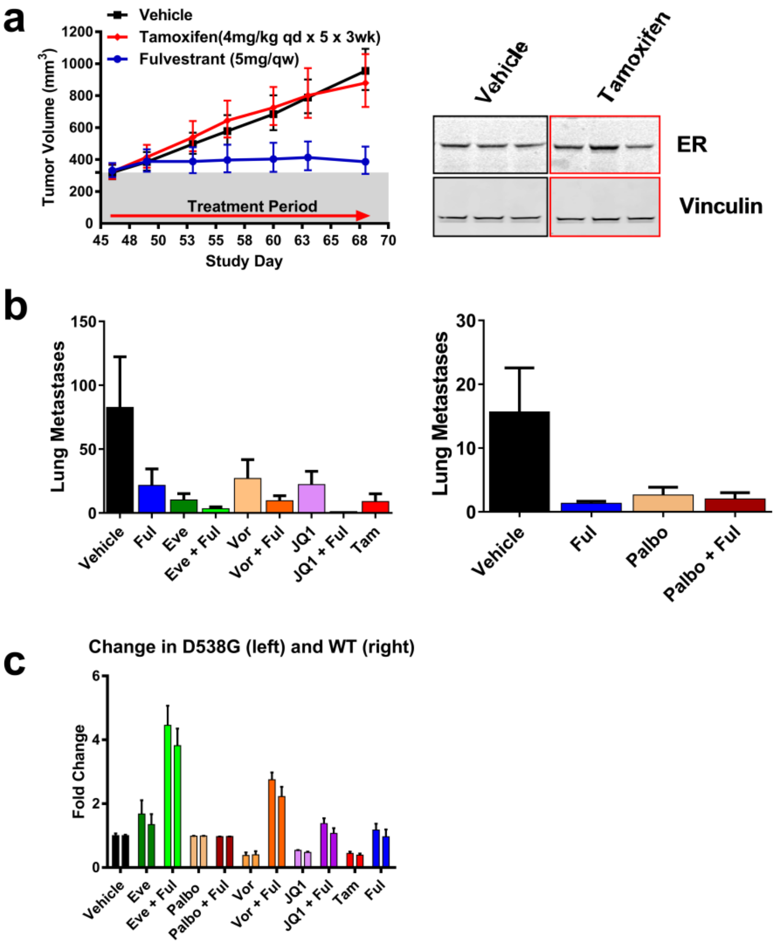

Supplementary Figure 2: (A) Efficacy of tamoxifen and fulvestrant. Vehicle and fulvestrant groups are the same as those represented in Fig. 3A-B, D. Western blot measuring ER from the 3 representative animals represented in A. Vinculin was used as a loading control. (B) Quantification of lung metastasis by counting visualized human specific DNA-PKcs staining. Each data point represents the average of 3 animals, where 3 random sagital cuts through the lung tissue were quantified. (C) Relative ER expression of the WT and D538G allele (represented as D538G/WT in Fig. 3F).

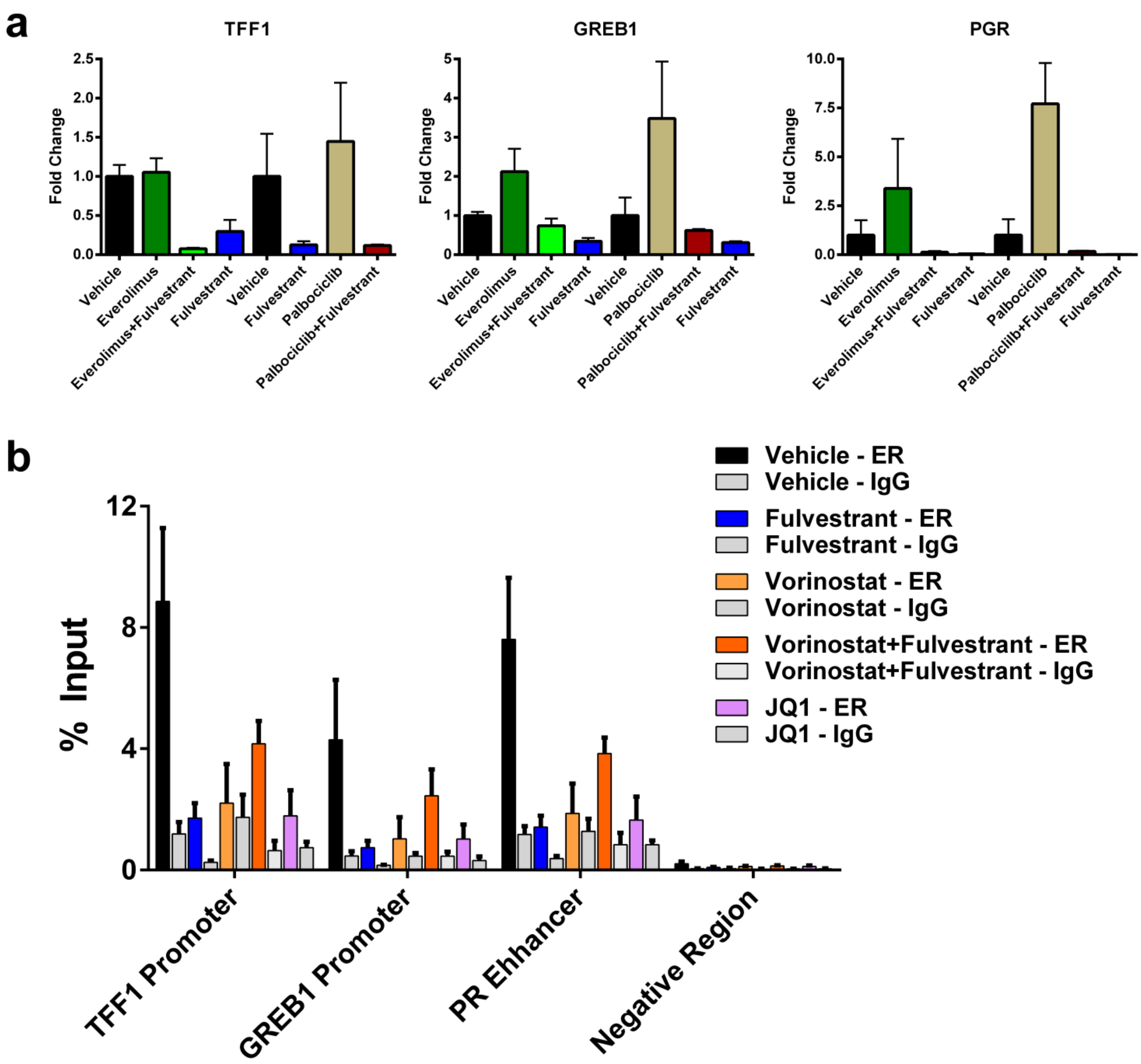

Supplementary Figure 3: (A) Gene expression of *TFF1* (left), *PR* (middle), and *GREB1* (right) from the same animals represented in Fig. 3C, 3D. Each point represents the average of 3 animals, bars represent SEM. Data demonstrates that everolimus or palbociclib alone to not decrease ER target gene expression. (B) Chromatin immunoprecipitations from Fig. 4C. Data includes IgG controls for each sample.

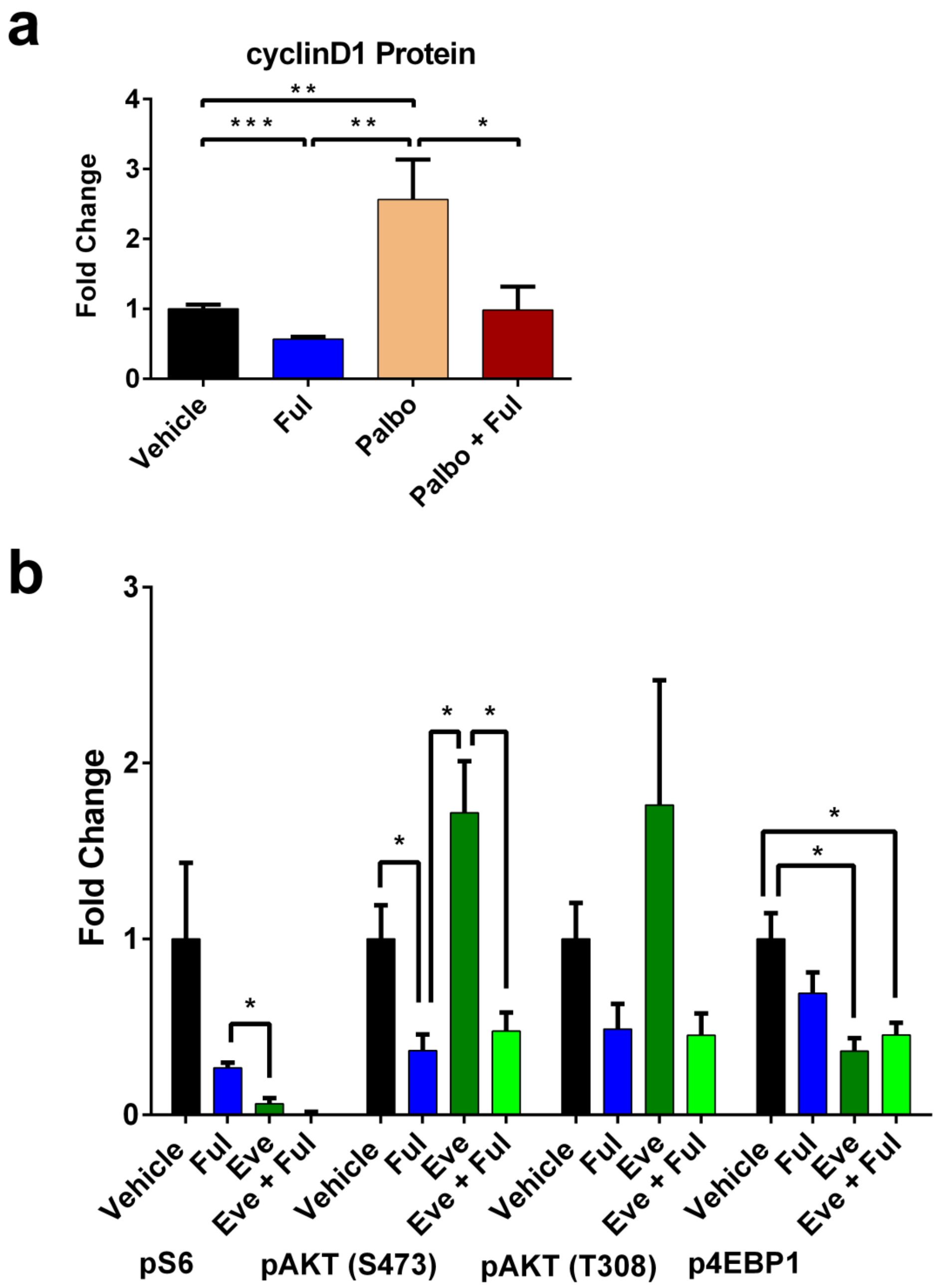

Supplementary Figure 4: (Goes with figure 5). (A-B) Quantification of the western blots represented in Fig. 5A/C, respectively. Bars indicate SEM. * indicates $p < 0.05$, ** indicates $p < 0.005$, *** indicates $p < 0.0005$. Significance was calculated using an unpaired two-tailed t test.

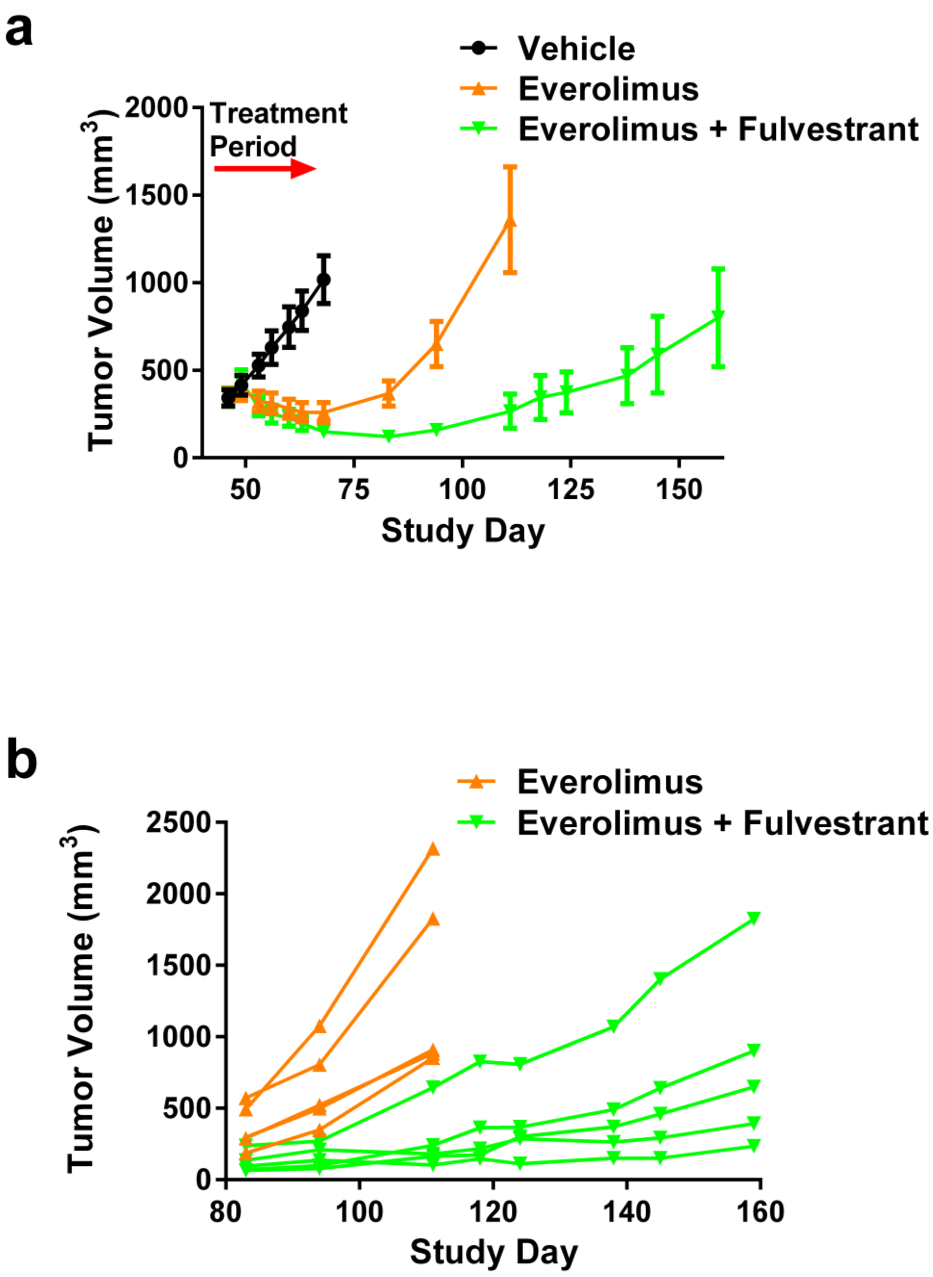

Supplementary Figure 5: (Goes with figure 6). (A) Animals from Fig. 3D were treated with everolimus alone or in combination with fulvestrant were allowed to regrow and are represented as in **Fig. 6**. (A) Mean tumor volume. Arrow represents 21 dosing day period. (B) Individual animal tumor growth.

a

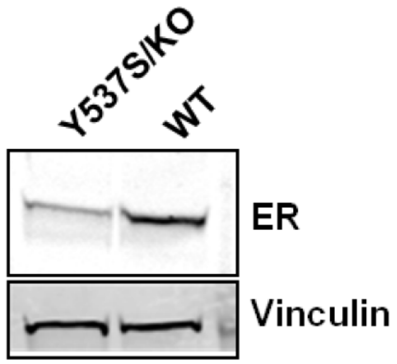

b

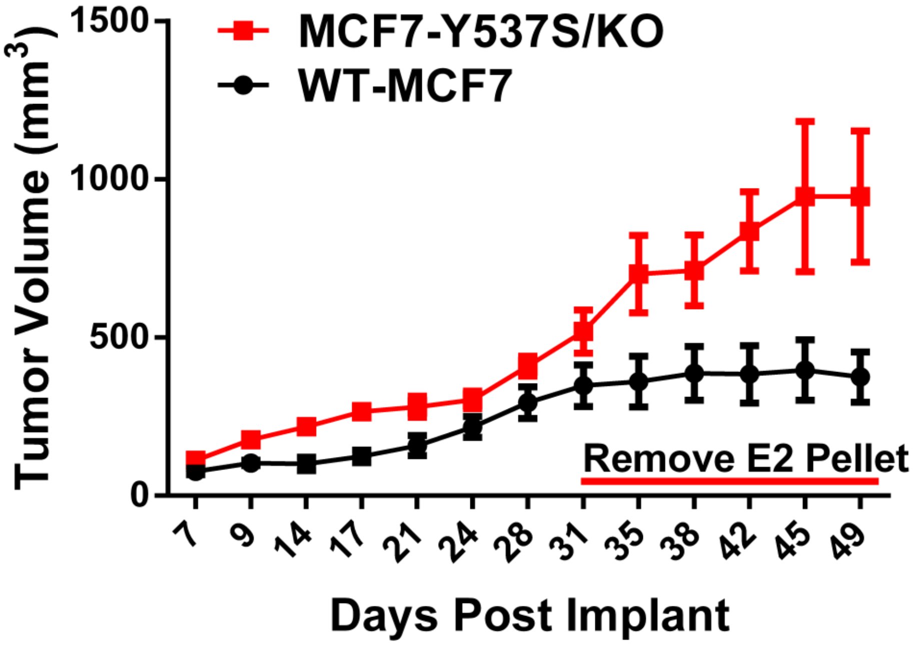

Supplementary Figure 6: (Goes with figure 7). (A) Western blot of ER in MCF7 parental and Y537S/KO cell lines. Data demonstrates lower ER expression in the Y537S/KO cell line. (B) Tumor growth of WT and Y537S/KO cells *in vivo*. Estrogen pellets were removed during exponential growth of at least four animals to demonstrate estrogen independent growth in the MCF7-Y537S/KO cell line, p=0.0264.

Supplementary Table 1: Table listing genetic features identified in CTC-174. All mutations identified by RNAseq were cross-referenced with COSMIC. *MYC* amplification was identified by CGH array.

Gene	Amino Acid Change	Allele Frequency
NBPF14	P920T	21%
RHBG	R425fs	31%
ESR1	D538G	31%
PIK3CA	N345K	38%
CASP8AP2	G237D	38%
PRAMEF11	R228H	40%
PPAP2B	R221Q	46%
SEC63	K529fs	50%
VTCN1	S192L	54%
MLL3	D348N	64%
ZNF792	E563K	64%
ADAMTS1	V652I	64%
MLL3	Y816fs	79%
POU5F1	E238Q	100%
IGLON5	R308C	100%
MYC	AMP	
